# Analysis of Permeation and Release Behavior Based on Structural Differences in the Gelatin Network within Hydrogels

**DOI:** 10.1002/mabi.202400628

**Published:** 2025-07-16

**Authors:** Tamaki Maeda, Satsuki Tajima, Miho Suto, Kazuki Murai

**Affiliations:** ^1^ Department of Chemistry and Materials Faculty of Textile Science and Technology Shinshu University Ueda Nagano Japan

**Keywords:** anisotropic polymer structure, bioinspired materials, dual drug delivery system, gelatin hydrogel, molecular permeability

## Abstract

Living organisms exhibit unique functionalities through reversible structural transitions of biomacromolecular assemblies, enabling molecular recognition and selective permeability. Inspired by these systems, we investigated anisotropic and isotropic gelatin hydrogels as models to mimic the structural transitions of biological channels. Using a template‐based method, anisotropic gelatin networks were formed with polypropylene and polyvinyl chloride templates, while isotropic networks were fabricated on glass substrates. Permeability studies with model molecules (phenylalanine, methylene blue, and rhodamine B) demonstrated that molecular properties (compound's balance between hydrophobicity and hydrophilicity) influenced transport behaviors, highlighting structural dependency. Mineralization experiments further validated the hydrophobic regions within anisotropic hydrogels, promoting silica formation while restricting calcium phosphate deposition. Additionally, drug release studies indicated anisotropic hydrogels preferentially released hydrophobic molecules, while isotropic hydrogels favored hydrophilic drugs. These findings elucidate the role of network anisotropy in the functions of hydrogel and provide insights into designing bioinspired functional materials for applications such as drug delivery systems and biomimetic membranes.

## Graphical abstract

This study investigates the impact of anisotropic gelatin network formation in hydrogels on molecular permeability, mineralization behavior, and drug release. Anisotropic networks, formed using polypropylene and polyvinyl chloride templates, create hydrophobic regions favoring hydrophobic molecule permeability and silica mineralization, while inhibiting hydrophilic ion transport. These hydrogels enable controlled dual‐drug release systems via molecular properties based on drug hydrophobicity.

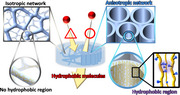

## 1 Introduction

Biological systems achieve various essential life functions by reversibly and cooperatively modulating the 3D ordered structures of molecular assemblies composed of multiple biomacromolecules. In particular, soft tissues (e.g., cartilage and organs) consist of a small amount of biomacromolecules and a large proportion of water. It is well known that these tissues exhibit high functionality through the control of the orientation and network structures of self‐assembling biomacromolecules [1, 2, 3]. For example, cartilage demonstrates unique mechanical properties due to its collagen‐based network, which exhibits distinct orientations and network structures at different hierarchical levels [4, 5]. Similarly, ion channels within cell membranes recognize external factors such as proton concentration, potential differences, and binding to specific biomolecules, and exhibit selective permeability to ions and molecules through reversible structural changes in their 3D conformation [6, 7]. These biological functions suggest that biomolecular complexes within living systems utilize reversible structural transitions in response to environmental changes as a mechanism for functional expression. Therefore, research on the structural control of soft materials, particularly hydrogels, which exhibit structural transitions in response to external stimuli, not only provides critical insights into the functional mechanisms of biomolecular assemblies but also contributes to the development of next‐generation soft materials that mimic biological functions. Hydrogels are representative soft materials that retain water within a minimal polymer network, thereby exhibiting a composition similar to that of biological soft tissues [8, 9, 10, 11]. However, the key difference between biological soft tissues and hydrogels lies in the presence of hierarchical and ordered polymer network structures. While biological soft tissues achieve unique functionalities by precisely controlling the hierarchical and ordered structures of biomacromolecular networks, current hydrogels lack such a level of structural precision. Hydrogel materials have been widely explored for applications such as drug release and cellular uptake, making them promising candidates for drug delivery system (DDS) matrices [12, 13]. However, controlling these properties requires the molecular‐level design of the polymer network structure within hydrogels, making it challenging to achieve functional regulation using identical material components. As a result, conventional hydrogels are not ideal for discussing the mechanisms by which structural differences in polymer networks alone lead to functional expression.

**FIGURE 1:**
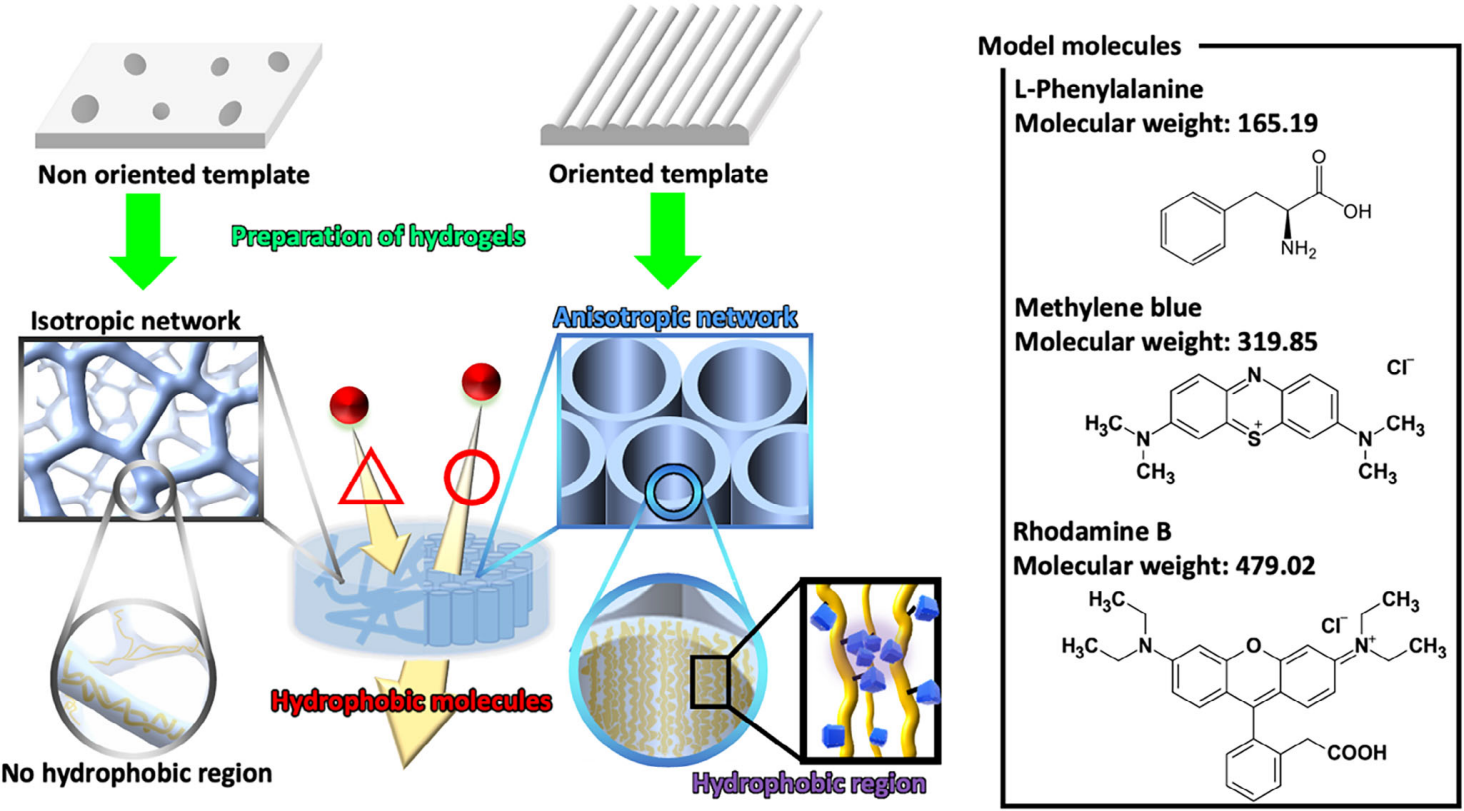
Schematic illustration of molecular permeation properties induced by structural differences in the gelatin network within the hydrogels. The molecular structure and molecular weight of model molecules were summarized in this Figure.

In our previous research, we demonstrated that hydrogels can be fabricated with anisotropic and isotropic gelatin networks by forming them on ordered and disordered templates, respectively [14, 15]. This approach mimics the structural control mechanisms observed in biomineralization, where biological molecules and inorganic materials achieve ordered structural formation. Hydrogels with such structurally controlled gelatin networks exhibit distinct mechanical properties and swelling behaviors depending on their network structures. For example, compression resistance along the orientation axis of the gelatin network and selective swelling perpendicular to the orientation axis were observed. This method enables the construction of both anisotropic and isotropic structures within a network composed of identical biomacromolecules, providing an effective means of evaluating the mechanical properties of hydrogels based solely on structural differences. Thus, we considered that hydrogels composed of the same biomacromolecular components (same type of biomacromolecule and water content) serve as ideal materials for elucidating the correlation between polymer network structural differences and functional expression mechanisms, which have remained a black box in conventional hydrogel research. The objective of this study is to analyze how the structural differences between anisotropic and isotropic gelatin networks, controlled via a template method, influence the permeation and release behaviors of model drugs. Figure 1 illustrates the conceptual diagram of the material permeation and release behavior induced by the structurally controlled gelatin networks within hydrogels. The most distinctive feature of this study is the analysis of drug permeation and release properties using hydrogels that, despite having identical composition (polymer network type and water content), exhibit clear structural differences at both the molecular and molecular‐assembled scales. This approach allows us to infer causal relationships between hydrogel functionalities—such as molecular permeation and release properties—and the polymer network structures of the hydrogel matrix. Consequently, the fundamental insights gained from this study are expected not only to clarify the impact of hydrogel network structural design on material permeation properties but also to contribute to the design principles of functional hydrogels that mimic biological systems utilizing reversible structural transitions. Moreover, this research will enhance the scientific understanding of function expression mechanisms in biological systems based on reversible structural transitions.

## 2 Results and Discussion

### 2.1 Analysis of the Influence on Molecular Permeability Behavior Based on Anisotropic Network Formation

**FIGURE 2:**
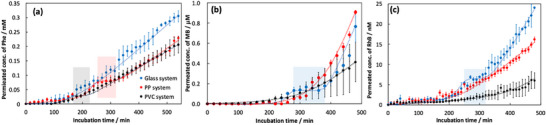
Permeation behavior of (a) Phe, (b) MB, and (c) RhB molecules on the anisotropic (PP systems: red circles and dotted line and PVC system: black circles and dotted line) and isotropic hydrogels (blue circles and dotted line). The colored bands indicate the regions corresponding to the induction phase observed in the oriented and non‐oriented systems.

We selected polypropylene (PP) and polyvinyl chloride (PVC) sheets as an orientational template to induce the anisotropic growth of the gelatin network within the hydrogel. On the other hand, the isotropic gelatin network hydrogels were fabricated using a glass substrate (an unoriented template). The disk‐shaped hydrogels equilibrated in swelling displayed a clear, transparent appearance for both systems (using the orientational and unoriented templates), yet exhibited distinctly different gelatin network morphologies (Figure S1). Scanning electron microscopy (SEM) observation revealed that hydrogels prepared with the PP and PVC templates had an anisotropic gelatin network structure oriented perpendicular to the disk surface. In contrast, the hydrogels prepared with the glass template observed identical morphology from any viewing direction, suggesting that they only exhibited isotropic gelatin network structures, unlike the PP and PVC systems. We investigated the permeability behavior of molecules through the hydrogels with differing gelatin network structures (anisotropic or isotropic structures). The differences between anisotropic and isotropic gelatin network structures were seen as model systems representing the pre‐ and post‐transition states of a channel's structural transformation to achieve functionality. Permeability behavior in the anisotropic and isotropic hydrogels was assessed using three model molecules with different *n*‐octanol/water partition coefficients (logP): L‐phenylalanine (Phe, logP = –1.5) [16], methylene blue (MB, logP = –0.1) [17], and rhodamine B (RhB, logP = 2.3) [18]. The logP value serves as an index of a compound's balance between hydrophobicity and hydrophilicity [19] and is defined by Equation (1):

(1) $$\mathrm{logP}=\log\frac{{\left[C\right]}_{n-\mathrm{Octanol}}}{{\left[C\right]}_{\mathrm{Water}}}$$

where [C]*_n_*_‐Octanol_ and [C]_Water_ represent the molar concentration of the compound in *n*‐octanol or water phases. Thus, logP > 0 indicates a hydrophobic compound, while logP < 0 indicates a hydrophilic compound. Moreover, the molecular structure and molecular weight of model molecules were summarized in Figure 1. Figure 2 shows the permeability behavior of each hydrogel for the selected model molecules. We observed permeability for all model molecules in both the anisotropic and isotropic gelatin hydrogel systems. However, the permeability behavior differed between the anisotropic and isotropic hydrogels. Specifically, for Phe, which is the most hydrophilic among the model molecules, the hydrogel with the isotropic gelatin network (Glass system) exhibited an induction phase with low permeability at the initial incubation period but eventually displayed typical diffusion behavior [20, 21]. In contrast, the hydrogels with an anisotropic gelatin network (PP and PVC systems) showed a unique permeability behavior different from typical diffusion, with two induction phases and two permeation phases. Furthermore, permeability behavior through the hydrogel for MB and RhB, which have larger logP values than Phe, contrasted with that for Phe. Specifically, the hydrogels with an anisotropic gelatin network displayed typical diffusion behavior, whereas the hydrogel with an isotropic gelatin network showed unique permeability behavior, including an induction phase and two permeation phases. The hydrogels used in this experiment had the same molecular composition and water content; the only differing factor was the anisotropy of the gelatin network. Therefore, these results suggest that the presence or absence of anisotropy in the gelatin network structure within the hydrogel influences the permeability behavior of the model molecules based on their molecular properties, specifically their hydrophobicity. In the Glass system, the hydrogel network, composed of gelatin (a water‐soluble protein), displayed a high affinity with the most hydrophilic molecule, Phe, among the three model molecules, facilitating efficient molecule transport via concentration gradients into and through the gel, likely resulting in diffusion behavior. In contrast, for MB and RhB, which are more hydrophobic than Phe, transport via concentration gradient into the hydrogel's hydrophilic environment was more challenging, leading to unique permeability behavior. However, in the anisotropic PP and PVC systems, the hydrogel showed favorable permeability behavior for hydrophobic molecules like MB and RhB, which suggests that the anisotropic gelatin network structure formed using the orientational templates (PP and PVC sheets) created a hydrophobic environment, favoring interaction with hydrophobic molecules. In our previous studies, we reported that hydrogels formed with the PP template establish their network on a gelatin film, initiated by phase separation on the hydrophobic PP surface during the gelation process [14]. Consequently, to form favorable interactions with the PP template, gelatin molecules may partially create hydrophobic regions dense with hydrophobic amino acids, leading to an environment more hydrophobic than the Glass system. As a result, the hydrogel with an anisotropic gelatin network exhibited efficient molecular transport and permeability behavior for hydrophobic molecules like MB and RhB via concentration gradients, in contrast to the hydrophilic molecule Phe. On the other hand, in the PVC system, the weaker interaction between the PVC template and gelatin molecules compared to the PP system may have limited the formation of large‐scale hydrophobic region formation within the hydrogel. Consequently, the hydrogel prepared using the PVC system likely did not exhibit higher permeability toward hydrophobic molecules, as observed in the anisotropic hydrogel (PP system). Additionally, the hydrogel formed in the PVC system possesses anisotropic tubular networks aligned parallel to the disk gel surface. Therefore, the gelatin network may act as a physical barrier to the model molecules, leading to lower permeability than that of the anisotropic hydrogel prepared using the PP system. Indeed, the permeability of the hydrogel prepared using the PVC system decreased with increasing molecular size of the model compounds, Phe, MB, and RhB.

### 2.2 Verification of Hydrophobic Region Formation using CaP and Silica Mineralization

**FIGURE 3:**
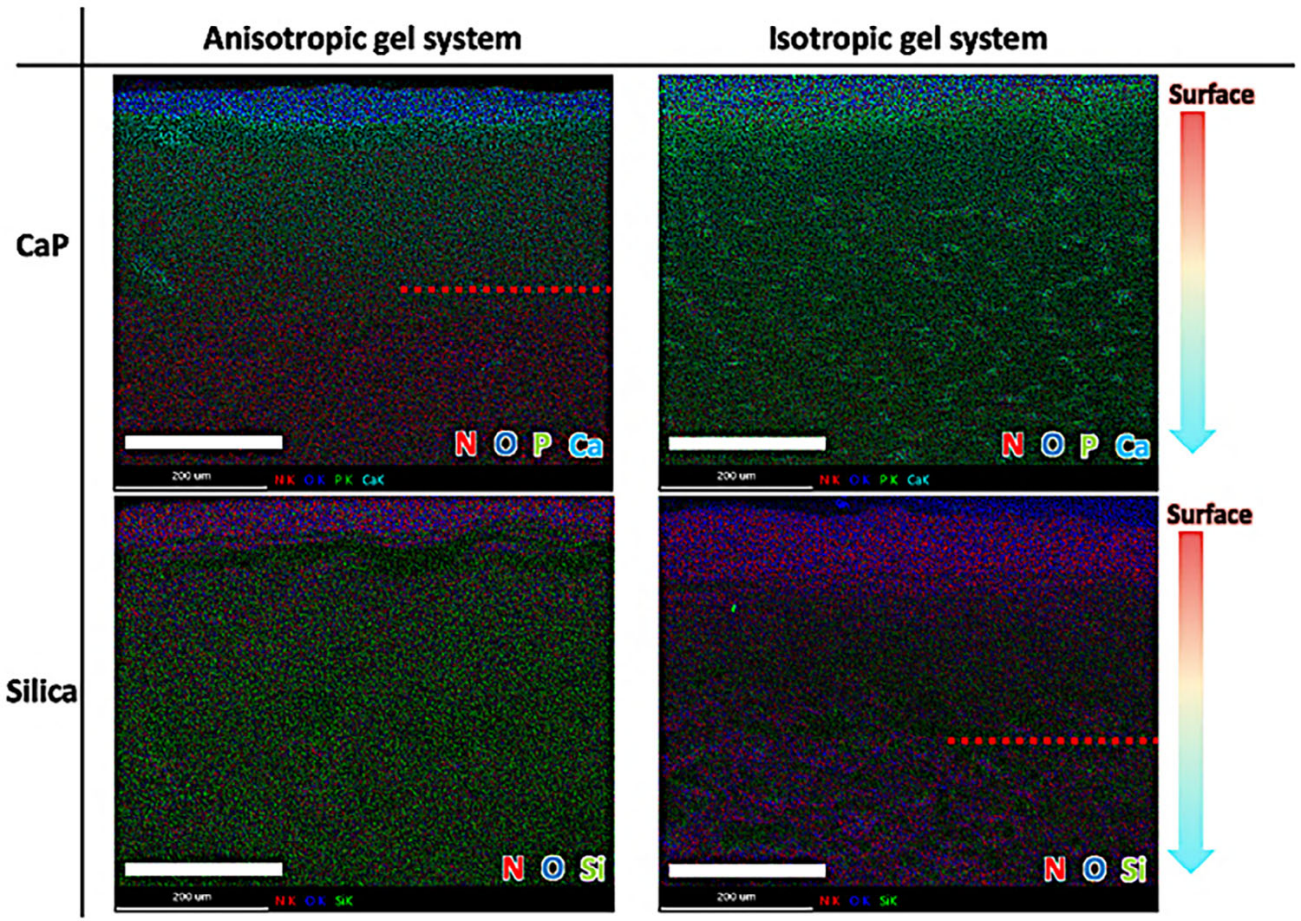
EDX mapping images of CaP‐ and silica‐mineralized hydrogels with anisotropic and isotropic gelatin networks. The red dashed lines in the images indicate the interface between the regions with and without inorganic material formation (CaP or silica). The scale bars are 200 µm.

To confirm the formation of hydrophobic regions within a hydrogel containing the anisotropic gelatin network, we analyzed the mineralized regions within the hydrogel using mineral sources with varying degrees of hydrophilicity. We selected SEM and EDX mapping images to observe the distribution of inorganic materials formed during mineralization. In this process, silica mineralization was performed using tetraethoxysilane (TEOS), a precursor with hydrophobic properties (theoretical prediction of logP = 5.10) [22], while calcium phosphate (CaP) was formed using calcium chloride and diammonium hydrogen phosphate as mineral sources. If hydrophobic regions have been established inside the anisotropic hydrogel through the self‐assembly of gelatin molecules, we predicted that CaP mineralization, using hydrophilic ions, would only mineralize on the hydrogel surface and not in the interior. In contrast, silica mineralization using the hydrophobic precursor TEOS would occur on both the interior and surface of the hydrogel. Figure 3 and Figure S2 present the EDX mapping images and SEM images of CaP and silica mineralized on either anisotropic or isotropic gelatin hydrogels, showing the cross‐sectional sections of these mineralized regions. These EDX and SEM images demonstrate clear differences depending on the type of hydrogel scaffold used for mineralization, as well as the selected mineral source. In the CaP mineralization system, the isotropic gelatin network hydrogel (Glass system) showed Ca mapping images supporting CaP formation throughout all cross‐sectional regions of the hydrogel, and SEM observations confirmed CaP mineralization in both surface and internal regions of the hydrogel. In contrast, the anisotropic gelatin network hydrogel (PP system) showed Ca mapping images supporting CaP formation only in regions near the hydrogel surface, and SEM observations revealed no CaP mineralization within the internal regions of the hydrogel. This suggests that CaP formation via mineralization occurs only in regions close to the surface of the anisotropic hydrogel, and does not occur within the interior regions. In the silica mineralization system, different results were observed compared to the CaP mineralization system [23]. Specifically, the isotropic gelatin network hydrogel (Glass system) showed Si mapping images supporting silica formation only in regions near the hydrogel surface, and SEM observations confirmed that silica mineralization did not occur in the internal regions of the hydrogel. Conversely, in the anisotropic gelatin network hydrogel (PP system), Si mapping images and SEM images were observed supporting silica formation throughout all cross‐sectional regions of the hydrogel. Thus, silica formation through mineralization occurs only in regions near the surface of isotropic hydrogels and does not occur in the internal regions of the hydrogel. These results align with hypotheses predicted through permeability experiments using Phe, MB, and RhB, and support that hydrophobic regions were established within the hydrogel via the formation of an anisotropic gelatin network structure. Consequently, in the PP system with internal hydrophobic regions established by an anisotropic gelatin network structure, the hydrophobic precursor (TEOS) was efficiently transported into the hydrogel interior, whereas CaP mineralization using a hydrophilic mineral source was hindered due to the internal hydrophobic regions of the hydrogel. Therefore, CaP mineralization resulted in CaP formation only at the hydrogel surface, while silica mineralization formed silica not only on the hydrogel surface but also within the internal regions.

### 2.3 Application as a Drug Delivery System Carrier

**FIGURE 4:**
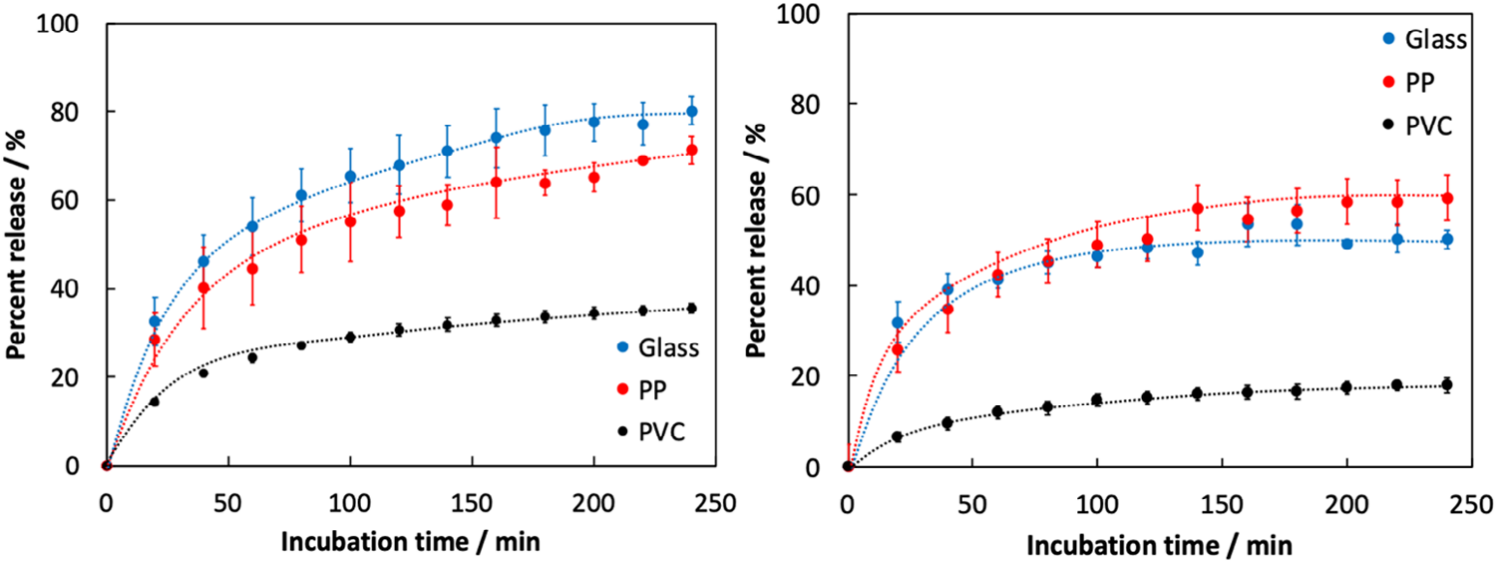
Drug release behaviors of (a) MB and b) RhB from the anisotropic (PP systems: red circles and dotted line and PVC system: black circles and dotted line) and isotropic hydrogels (blue circles and dotted line) in 50 mM of phosphate buffer solution (pH 7.4). The release test was carried out three times.

Finally, we investigated the potential of gelatin hydrogels with controlled structures as carriers for drug delivery systems. Specifically, we analyzed how the presence of hydrophobic regions formed within the anisotropic gelatin hydrogel influenced the release behavior of loaded model drugs. For the drug release tests, MB as a hydrophilic molecule, and RhB as a hydrophobic molecule, were selected as model drugs. Figure 4 shows the release behavior of MB or RhB molecules from anisotropic or isotropic gelatin hydrogels loaded with a single drug. We used the percent release (%R) to evaluate the release behavior of the model drug from the hydrogel. The percent release was defined as the ratio of the amount of the model drug released from the hydrogel at a given time to the total amount initially loaded in the hydrogel and was estimated using Equation (2):

(2) $$\%R\left[\%\right]=\frac{C_{R}\left[mM\right]}{C_{L}\left[mM\right]}\times 100$$

In this equation, C_R_ and C_L_ represent the concentration of the model drug released from the hydrogel at a given time and the total concentration of the model drug initially loaded in the hydrogel, respectively. First, we analyzed the loading efficiency of model drugs into the anisotropic or isotropic hydrogels prepared in each system, and the results are summarized in Table 1. In all systems, the amount of the hydrophilic model drug MB loaded into the hydrogels was higher than that of the hydrophobic model drug RhB. Moreover, the amount of each model compound loaded was generally consistent across different hydrogel types, suggesting that the specific gelatin network structures within the hydrogels did not significantly affect drug loading. On the other hand, the release of both MB and RhB molecules from the hydrogels showed behavior consistent with molecular diffusion at all systems. Interestingly, the percent release of the loaded drugs varied depending on the type of hydrogel carrier. Specifically, in the case of hydrogels with an anisotropic gelatin network (PP system), a higher percent release was observed for RhB, a hydrophobic molecule, compared to the isotropic gelatin network hydrogels (Glass system). Conversely, for the Glass system, a higher percent release was observed for MB, a hydrophilic molecule, compared to the PP system. For the hydrophilic molecule MB, the percent release after 240 min was 80.1 ± 3.2% for the isotropic hydrogel (Glass system) and 71.3 ± 3.1% for the anisotropic hydrogels (PP system). Additionally, in the late incubation stage (after 180 min), there was no overlap in the error bars based on the standard deviation. On the other hand, for the hydrophobic molecule RhB, the percent release after 240 min was 50.1 ± 2.0% for the isotropic hydrogel (Glass system) and 59.3 ± 1.2% for the anisotropic hydrogels (PP system). However, we found that the percent release of each model drug from the anisotropic hydrogels with gelatin networks prepared using the PVC template was significantly lower compared to those from anisotropic or isotropic hydrogels prepared using other systems (MB system: 35.6 ± 1.1% and RhB system: 18.0 ± 1.5%). These results support the conclusion that the release behavior of the model drugs differs depending on the type of hydrogel. These results can be explained as follows: in the anisotropic hydrogel (PP system), the formation of hydrophobic regions within the hydrogel likely inhibited the molecular diffusion of MB, a hydrophilic molecule, within the hydrogel, thereby reducing its release into the external environment. In contrast, RhB as a hydrophobic molecule was likely unaffected by the hydrophobic regions and could be efficiently released into the external environment. Also, the reduction of percent release observed in the anisotropic hydrogel (PVC system) compared to the other anisotropic hydrogel (PP system) can be explained from two perspectives: (1) the suppression of large‐scale hydrophobic region formation and (2) the presence of a physical barrier formed by the gelatin network aligned parallel to the disk surface of the hydrogel. As previously mentioned, the interaction between the PVC template and gelatin molecules is weaker than that in the PP system. Consequently, although the formation of anisotropic gelatin networks still occurs, the development of extensive hydrophobic domains is hindered. Additionally, the anisotropic gelatin network aligned parallel to the disk surface acts as a physical barrier, affecting the release of the model compounds loaded within the hydrogel. Therefore, compared to the PP‐based hydrogels, the PVC‐based hydrogels exhibited both reduced selective permeability and decreased release amounts. Thus, the differences in release rates of the model drugs from the hydrogels suggest that these behaviors were achieved through selective permeation enabled by the formation of an anisotropic gelatin network.

**TABLE 1:** Amount of model drugs into the anisotropic or isotropic hydrogels prepared in each system.

Hydrogel systems	Loaded model drugs		
	MB (Percent loading)	RhB (Percent loading)	MB / RhB (Percent loading)
Glass	37.7 ± 3.3 µm	6.4 ± 0.3 µm	336.7 ± 3.0 µm 6.3 ± 0.3 µm (73.3 ± 6.0%) (12.6 ± 0.7%)
	(75.5 ± 6.6%)	(12.9 ± 0.7%)	
PP	41.2 ± 1.1 µm	5.9 ± 0.3 µm	40.4 ± 0.3 µm 4.5 ± 0.0 µm (80.8 ± 0.6%) (8.9 ± 0.0%)
	(82.4 ± 2.2%)	(11.2 ± 0.7%)	
PVC	43.1 ± 0.3 µm	10.0 ± 0.3 µm	43.2 ± 0.2 µm 11.9 ± 0.1 µm (86.5 ± 0.2%) (23.7 ± 0.2%)
	(86.2 ± 0.5%)	(19.9 ± 0.7%)	

**FIGURE 5:**
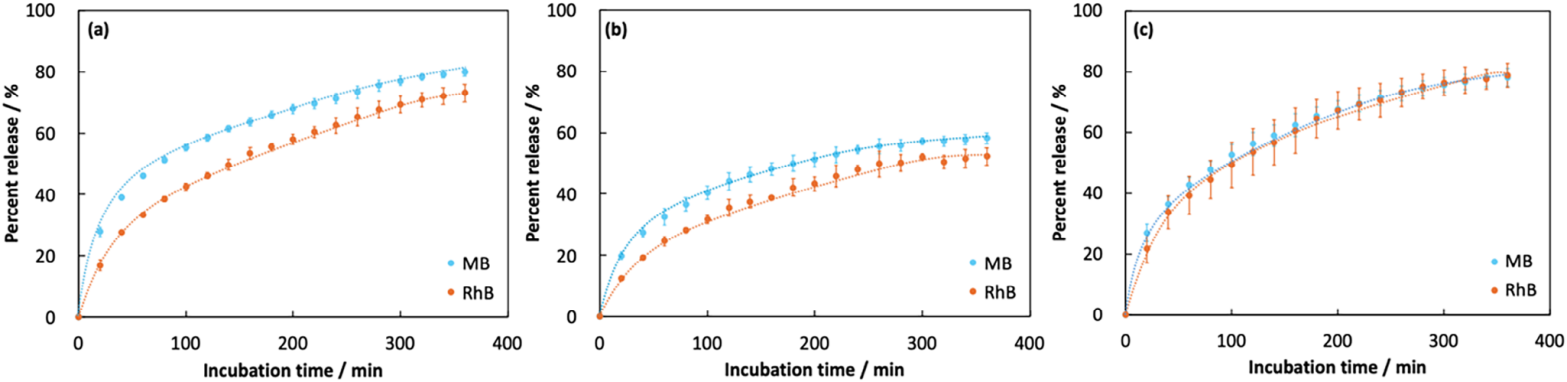
Dual drug release profiles from the anisotropic ((a) PP and (b) PVC systems) and (c) isotropic gelatine hydrogels (Glass system) in phosphate buffer solution (pH 7.4, 50 mm). The release test was carried out three times.

Based on the distinct release behaviors of drugs with different molecular properties from gelatin hydrogels, we demonstrated the potential of hydrogels loaded with two drugs of differing hydrophobicity (MB and RhB) as carriers for dual drug delivery systems. First, we analyzed the loading amounts of each drug into anisotropic and isotropic hydrogels using an aqueous solution containing both a hydrophilic drug (MB, 50 µM) and a hydrophobic drug (RhB, 50 µm) (Table 1). The loading amounts of each model drug into the hydrogels were similar to those observed when using single‐drug solutions, indicating that the simultaneous use of the two model drugs had no significant effect on drug loading. Figure 5 shows the release profiles of each drug from hydrogels loaded with both drugs simultaneously. We confirmed that drug release from the gelatin hydrogels was consistent with molecular diffusion, similar to the behavior observed for hydrogels loaded with a single model drug. However, focusing on the release behavior of MB and RhB molecules loaded into the gelatin hydrogels, we observed clear differences. In isotropic gelatin hydrogels (Glass system), the release behaviors (release percent and release rate) of MB, a hydrophilic molecule, and RhB, a hydrophobic molecule, were identical. In contrast, in the PP and PVC systems, MB molecules exhibited a higher release percent and faster release rate than RhB molecules. The primary reason why the release behavior in the dual drug‐loaded system exhibited an opposite trend compared to the single drug system is likely due to differences in drug‐loading characteristics of the hydrogels when both RhB and MB were simultaneously incorporated. The amount of each drug released from the anisotropic hydrogel in the dual‐drug system was generally consistent with that observed in the single‐drug system. In contrast, in the isotropic hydrogel, the amount of RhB released increased compared to the single‐drug‐loaded system. This result suggests that, in the isotropic hydrogel, co‐loading of the hydrophobic RhB with the hydrophilic MB destabilized the retention of RhB, causing it to be released more rapidly. Supporting this hypothesis, the amount of MB released from the isotropic hydrogel in the dual‐drug system was slightly higher than that in the single‐drug system. This increase may be attributed to a slight increase in the hydrophobicity of the hydrogel due to the simultaneous loading of RhB and MB, which destabilized MB retention and facilitated its release. Moreover, these results strongly support that the hydrophobic regions formed within the gelatin hydrogel contribute to distinctive release behaviors driven by differences in the molecular properties of the loaded dual drugs.

## 3 Conclusion

In conclusion, this study demonstrates the impact of anisotropic and isotropic gelatin network structures on molecular permeability, mineralization behavior, and drug delivery properties. By employing a simple template‐based fabrication method, we successfully controlled the anisotropic and isotropic structures within gelatin hydrogels and systematically analyzed their influence on functional behavior. The anisotropic gelatin network hydrogels exhibited unique molecular permeability patterns, enhanced interactions with hydrophobic molecules, and selective mineralization using hydrophobic precursors. These findings confirm the formation of hydrophobic regions within anisotropic hydrogels, which critically influenced their functional properties. Furthermore, drug release studies revealed that hydrogels with anisotropic gelatin networks can facilitate selective permeation, selectively releasing hydrophobic or hydrophilic drugs based on the network structure. These results underscore the potential of anisotropic gelatin hydrogels as biomimetic materials capable of mimicking biological functions such as selective permeability. This work provides valuable insights into the design principles for advanced hydrogel‐based materials with hierarchical and ordered structures. These findings pave the way for developing next‐generation soft materials that mimic the reversible structural transitions and functional expressions found in biological systems, with potential applications in drug delivery, tissue engineering, and bioinspired material design.

## 4 Experimental Section

### 4.1 Preparation of Anisotropic and Isotropic Gelatin Hydrogels

The anisotropic and isotropic gelatin hydrogels were prepared using a facile fabrication method according to the previous reports [14, 15]. First, gelatin derived from bovine skin (type B) was dispersed in ultrapure water and dissolved by heating the suspension in an incubator at 37°C. The concentration of the gelatin solution was fixed at 10 wt.%. The gelatin solution was introduced into a reaction cell, which was prepared by sandwiching two glass substrates (10 cm × 10 cm), separated by a 2.0 mm silicon spacer. To induce anisotropic growth of gelatin networks, polypropylene (PP) sheet‐coated and polyvinyl chloride (PVC) sheet‐coated glass substrates were used as an oriented template for structural control. Hydrogel formation was achieved by cooling the reaction cell filled with gelatin solution at 4°C. The resulting sheet‐shaped hydrogels were carefully removed from the reaction cell and cut into disk‐shaped hydrogels (diameter [*d*] × thickness [*z*]: 10.00 mm × 2.00 mm). The as‐prepared hydrogels were immersed in cold water for equilibrium swelling and stored in cold water until further measurements.

### 4.2 Molecular Permeation Studies of the Gelatin Hydrogels

The molecular permeation properties of the gelatin hydrogels were evaluated by examining the permeation behavior of three different molecules through the hydrogels. The selected model molecules were L‐phenylalanine (Phe), methylene blue (MB), and rhodamine B (RhB), which had different hydrophobicities defined by their *n*‐octanol‐water partition coefficient (logP). The disk‐shaped gelatin hydrogels (diameter [*d*] × thickness [*z*]: 10.00 mm × 2.00 mm) were placed in a 3 mm diameter hole at the bottom of the upper Petri dish, and 4 mL of solutions of the model molecules (Phe system: 50 mm, MB system: 2.0 mm, and RhB system: 2.0 mm) were slowly added into the upper Petri dish. The upper dish was set on top of a bottom dish filled with 12 mL of ultrapure water (Figure S3) and incubated at 4°C. The amount of molecules permeating through the hydrogel was determined by measuring absorbance at the maximum absorption wavelengths for each molecule (Phe: 257 nm, MB: 664 nm, and RhB: 554 nm) in the bottom dish at 20‐min intervals (MB system) or 10‐min intervals (RhB system) or 15‐min intervals (Phe system).

### 4.3 CaP and Silica Mineralizations

CaP mineralization was carried out using an alternate dipping method with 50 mm calcium chloride aqueous solution (4 mL) and 30 mm diammonium hydrogen phosphate aqueous solution (4 mL). First, the gelatin hydrogel (diameter [*d*] × thickness [*z*]: 10.00 mm × 2.00 mm) was immersed in the calcium chloride solution for 5 min, followed by washing with ultrapure water for 1 min. The calcium ion‐loaded gelatin hydrogels were then immersed in the diammonium hydrogen phosphate solution for 5 min and subsequently washed with ultrapure water for 1 min. This process was repeated five times to form CaP on the gelatin hydrogel through mineralization.

We carried out by silica mineralization according to the previous reports [23]. For silica mineralization, the gelatin hydrogel (diameter [*d*] × thickness [*z*]: 10.00 mm × 2.00 mm) was immersed in 4 mL of TEOS in a glass Petri dish for 24 h at 4°C. The resulting silica‐composite gelatin gels were washed with ultrapure water.

### 4.4 Drug Release Study from Gelatin Hydrogels

Drug loading and release studies were performed as follows: the hydrogels (diameter [*d*] × thickness [*z*]: 10.00 mm × 2.00 mm) were loaded with drugs by immersing them overnight in 4.0 mL of aqueous model drug solution (single system: 50 µm; dual system: each at 50 µm). MB and RhB molecules were selected as model dual drugs. Subsequently, the drug‐loaded gelatin hydrogels were washed with ultrapure water for 30 s. The amount of drug loaded into the hydrogel in each system was determined based on the absorbance of the remaining solution after incubation. Then, the gelatin hydrogels loaded with either a single drug or dual drugs were added to 4.0 mL of 50 mm phosphate buffer (pH 7.4). The drug‐loaded hydrogels were incubated in the buffer at 4°C and the amount of drug released from the hydrogel was determined by measuring absorbance in the 400–800 nm range. The drug release profile was estimated by calculating the percentage release, which is the molar ratio of the released drug to the initial drug loaded into the gelatin hydrogels.

### 4.5 Scanning Electron Microscopy Observation

The morphology of the gelatin networks, both at surface and cross‐sections, was observed using a scanning electron microscope (SEM, Hitachi S‐3000N) equipped with an energy‐dispersive X‐ray (EDX) spectrometer. SEM observations were performed on unstained samples at an accelerating voltage of 20 kV. The swollen hydrogels were cut to expose the surface and cross‐section (Figure S1) and then lyophilized. The dried gel samples were coated with platinum nanoparticles via ion sputtering.

## Conflicts of Interest

The authors declare no conflict of interest.

## Supporting information




**Supporting File 1**: mabi70039‐sup‐0001‐SuppMat.docx.

## Data Availability

Research data are not shared.
