# Supplementary material for: Analysis of Permeation and Release Behavior Based on Structural Differences in the Gelatin Network within Hydrogels
Source: Macromol Biosci. 2025 Jul 16;25(10):e00628. doi: 10.1002/mabi.202400628 (PMC12530698; doi:10.1002/mabi.202400628)
Supplement: Supplementary file 1 — Supporting File 1: mabi70039‐sup‐0001‐SuppMat.docx. [file MABI-25-e00628-s001.docx]

((Supporting Information can be included here using this template))

Supporting Information

Analysis of permeation and release behavior based on structural differences in the gelatin network within hydrogels

Tamaki Maeda, Satsuki Tajima, Miho Suto, and Kazuki Murai*

**Contents**

1. **Figure S1. Photographic and SEM images of the prepared gelatin hydrogels and schematic illustrations of anisotropic and isotropic gelatin networks.**
2. **Figure S2. SEM images of CaP and silica particles mineralized at the hydrogel interior with anisotropic and isotropic gelatin networks.**
3. **Figure S3. Schematic illustrations of device for the molecular permeation investigation.**


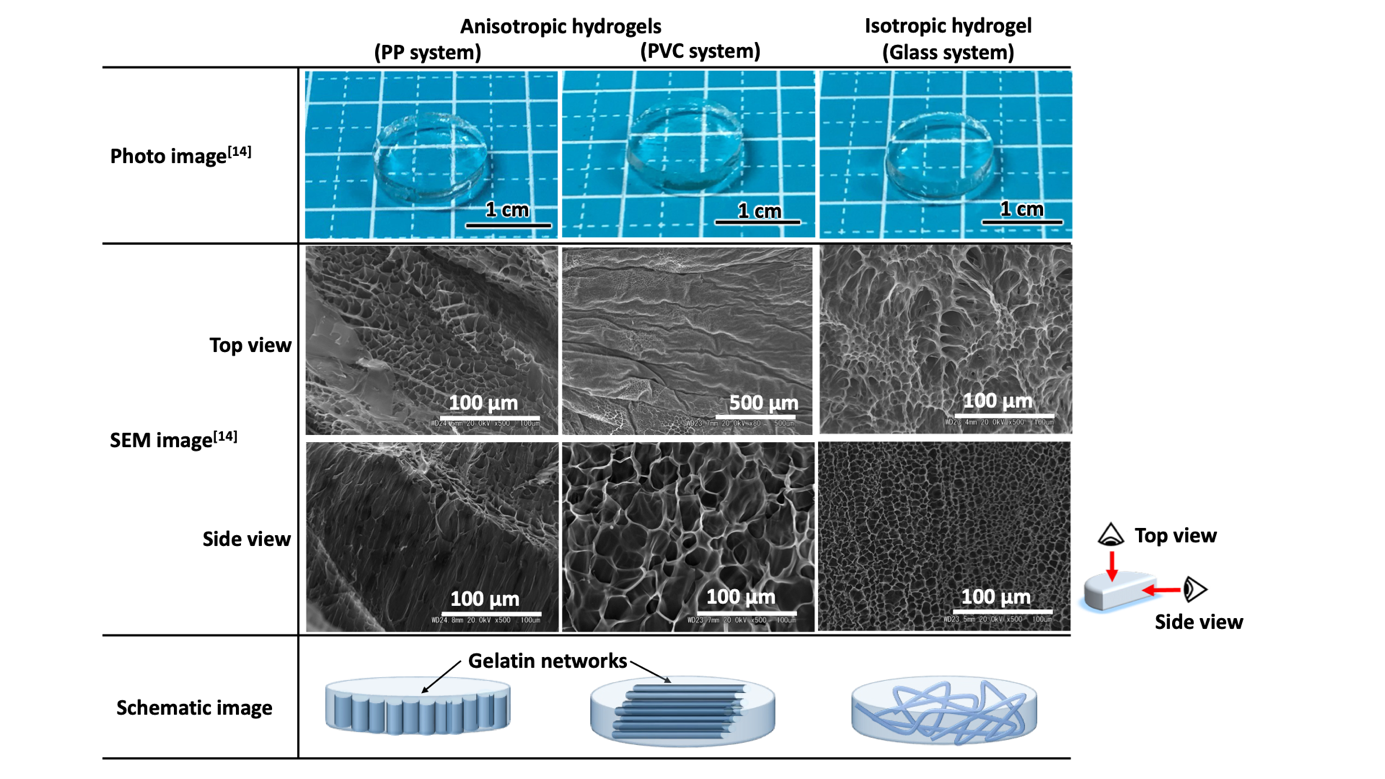


**Figure S1.** Photographic images of the prepared gelatin hydrogels, SEM images of the gelatin networks observed from top and side views, and schematic illustrations of anisotropic and isotropic gelatin networks. SEM and photographic images are reproduced from ref. [14] with permission from the Royal Society of Chemistry.

***
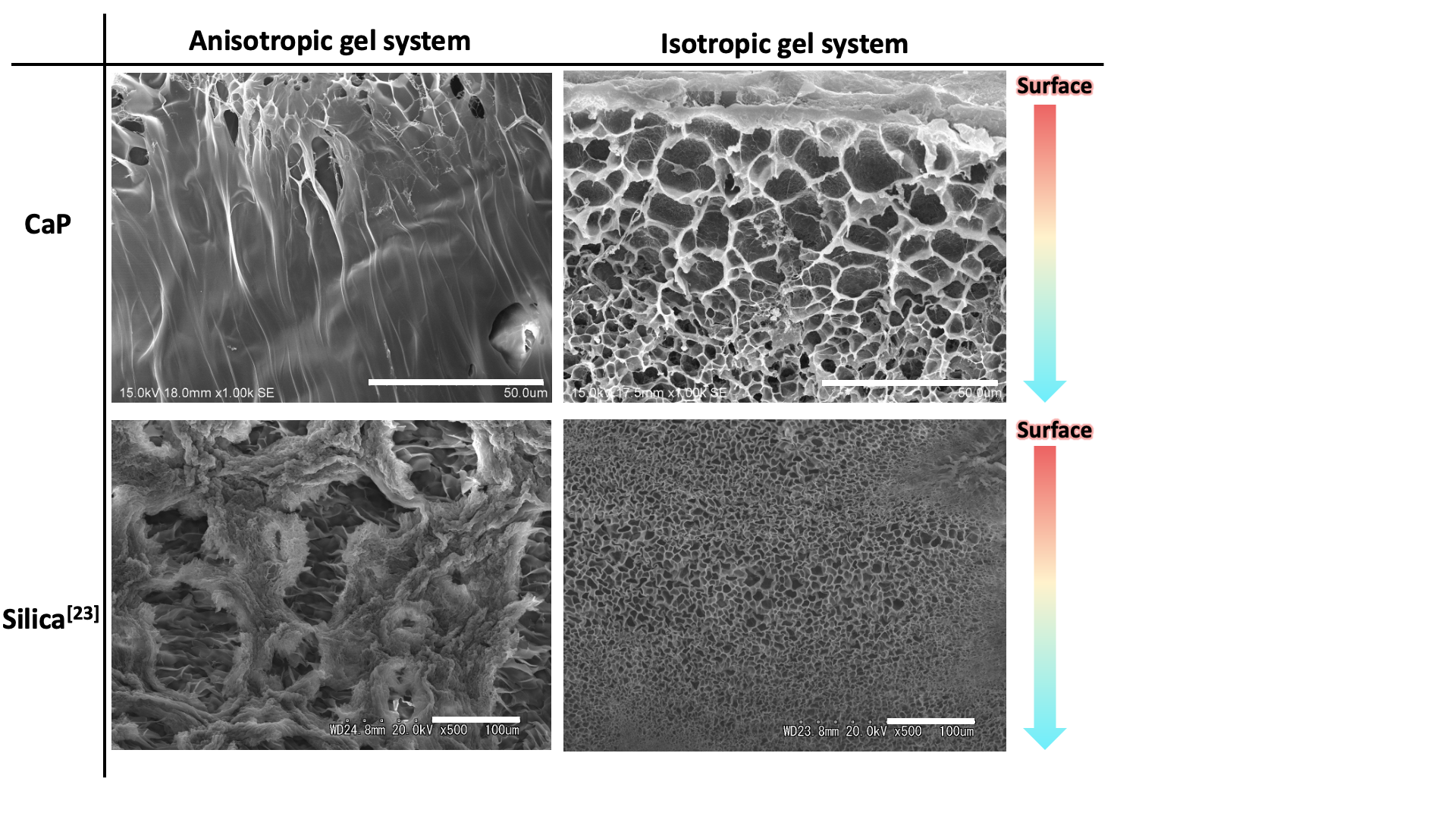
***

**Figure S2.** SEM images of CaP and silica particles mineralized at the hydrogel interior with anisotropic and isotropic gelatin networks. The scale bars are 50 μm. SEM images of silica mineralized at the anisotropic and isotropic systems reproduced from ref. [23] with permission from the Royal Society of Chemistry.


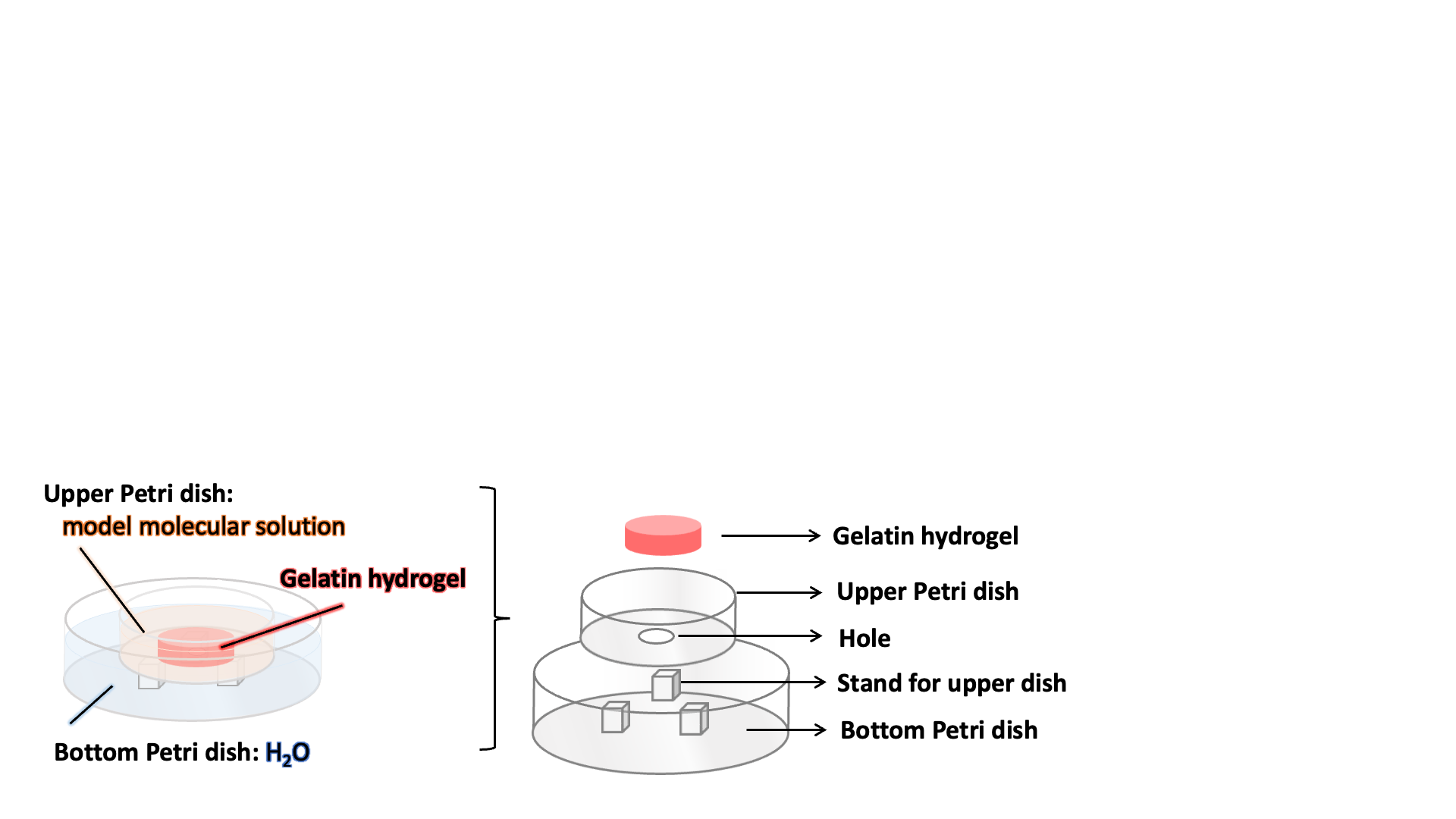


**Figure S3.** Schematic illustrations of device for the molecular permeation investigation.
